# Extreme Temperatures and Missed Pediatric Preventive Care Visits

**DOI:** 10.1001/jamanetworkopen.2026.10114

**Published:** 2026-04-27

**Authors:** Stephanie L. Mayne, Janani Ramachandran, Priyani Sharma, Joniqua Ceasar, Maura Powell, Brian P. Jenssen, Alexander G. Fiks

**Affiliations:** 1 Clinical Futures, Children’s Hospital of Philadelphia, Philadelphia, Pennsylvania; 2 Department of Pediatrics, University of Pennsylvania Perelman School of Medicine, Philadelphia; 3 Leonard Davis Institute for Healthcare Economics, University of Pennsylvania Perelman School of Medicine, Philadelphia; 4 PolicyLab, Children’s Hospital of Philadelphia, Philadelphia, Pennsylvania; 5 The Possibilities Project, Children’s Hospital of Philadelphia, Philadelphia, Pennsylvania

## Abstract

**Importance:**

Missed pediatric preventive care visits can delay essential developmental screenings, immunizations, and management of chronic conditions, adversely impacting child health. Understanding the relationship between extreme temperatures and missed preventive care is essential for developing adaptive strategies to maintain care access amid increasing climate variability.

**Objective:**

To assess the association of extreme temperatures with rates of missed pediatric preventive care visits and evaluate differences by patient age, insurance, and neighborhood socioeconomic status (SES).

**Design, Setting, and Participants:**

This cross-sectional time-series study used electronic health record data of patients aged younger than 19 years with scheduled preventive care visits at 32 primary care practices in the Children’s Hospital of Philadelphia primary care network in Pennsylvania and New Jersey from January 1, 2009, to December 31, 2023. Statistical analyses were completed between March and October 2025.

**Exposure:**

Daily maximum temperature (°F), derived from the National Oceanic and Atmospheric Administration’s weather stations and averaged at the county level for each practice.

**Main Outcome and Measures:**

Daily rate of missed preventive care visits (the proportion of scheduled visits resulting in same-day cancellation or no-show). Generalized linear quasi-Poisson models estimated associations of daily maximum temperature (in °F) with daily rate of missed preventive care visits overall and stratified by age, insurance, and neighborhood SES. Associations were evaluated separately for warm (May-October) and cold (November-April) months. Heterogeneity across strata was evaluated using the Cochran *Q* test.

**Results:**

Among 504 428 patients, 51% were male, 36% had public insurance, and 27% were from very low SES neighborhoods. Mean (SD) patient age was 6.0 (5.7) years. Among 4 137 542 scheduled preventive care visits, 13% were missed. Each 1 °F decrease below a maximum daily temperature of 41.5 °F in cold months and 1 °F increase above 88.0 °F in warm months was associated with a higher rate of missed visits (rate ratios, 1.01 [95% CI, 1.01-1.01] and 1.01 [95% CI, 1.00-1.01], respectively). Significant heterogeneity in missed visits across age, payer, and neighborhood SES was observed in cold months, with higher rates of missed visits among patients with commercial vs public or other types of insurance (RR, 1.02; 95% CI, 1.02-1.02) as well as among younger patients and those from high SES neighborhoods.

**Conclusions and Relevance:**

In this cross-sectional study, extreme outdoor temperatures were associated with increased rates of missed pediatric preventive care visits, with heterogeneity across age, payer, and neighborhood SES during cold months. The findings suggest proactive scheduling strategies and alternative care delivery models should be assessed to maintain access to essential pediatric preventive care services during temperature extremes.

## Key Points

**Question:**

Are extreme outdoor temperatures associated with rates of missed pediatric preventive care visits?

**Findings:**

In this cross-sectional study of 4 137 542 pediatric visits in a large primary care network, each 1 °F decrease below 41.5 °F in cold months and 1 °F increase above 88.0 °F in warm months was associated with a higher rate of missed visits. During cold months, rates of missed visits were higher among children who were younger, commercially insured, and from high socioeconomic status neighborhoods.

**Meaning:**

Extreme temperatures were associated with missed pediatric preventive care with differential associations across subgroups, suggesting a need for targeted interventions during temperature extremes.

## Teaser

This cross-sectional study investigates whether extreme hot or cold outdoor temperatures are associated with increased rates of missed preventive care visits in a pediatric population.

## Introduction

Global temperatures are expected to rise by 2.7 to 7.9 °F by the end of the 21st century.^1^ Temperature extremes pose a substantial threat to child health and well-being, through direct impacts such as heat-related illness and learning impairments and through potential impacts on clinical care.^2^ Extreme daily temperatures (ie, temperatures significantly hotter than average for a particular time and place) are increasingly frequent and have been shown to increase emergency department visits among children for heat-related illnesses and other conditions.^3,4,5^ However, less is known about the potential impact of extreme temperatures on the receipt of routine pediatric preventive care. Pediatric primary care is crucial to ensuring that children receive recommended preventive care such as vaccines and developmental screenings as well as management of chronic conditions and establishment of healthy life trajectories. High rates of no-shows, or scheduled but unattended primary care visits, adversely impact child health by delaying receipt of needed preventive care and negatively impact primary care practices’ resource utilization.^6,7,8^ Missed preventive care visits have multiple causes, including schedule conflicts and transportation issues.^9,10^ Extreme temperatures are a potential cause that has been understudied but which may become increasingly important as global temperatures rise.

A study of adult patients in Philadelphia reported that rates of missed primary care appointments increased by nearly a percentage point per 1 °F increase in daily maximum temperature above 89 °F or decrease below 39 °F.^11^ To date, little is known about the association of extreme temperatures, both hot and cold, with pediatric primary care visit attendance. The pediatric context may present particular challenges to visit attendance, such as dependence on caregivers and the necessity of working around school schedules. As such, a better understanding of whether extreme temperatures are associated with pediatric primary care visit attendance is needed.

In addition, although prior research indicates children from lower income populations have higher rates of missed primary care visits than higher income children,^12,13^ it is unknown whether extreme temperatures are differentially associated with missed preventive care visits among children of different ages and socioeconomic circumstances. Younger children may be more adversely affected by extreme temperatures,^4^ and caregivers might be more likely to reschedule visits for very young children on extremely hot or cold days. In addition, families experiencing lower socioeconomic status (SES), which might be proxied through insurance status or neighborhood socioeconomic circumstances, might be more impacted by weather due to greater reliance on public transportation. However, the existence and extent of such differences is unknown.

The objective of our study was to assess the association of extreme temperatures with the rate of missed preventive care visits in a large pediatric primary care network over a 15-year period and to determine whether any associations were moderated by patient age, insurance payer, and neighborhood SES. We hypothesized that the rate of missed preventive care visits would be higher on days with extreme temperatures (either cold or warm) and that the magnitude of increase would be higher among children younger than 2 years of age, those who were publicly insured, and those from low SES neighborhoods.

## Methods

### Study Design, Setting, and Population

In this cross-sectional study, we used an observational time series design to estimate associations of daily temperatures with rates of missed pediatric primary care appointments. The study was conducted within the Pediatric Research Consortium, the Children’s Hospital of Philadelphia (CHOP) primary care practice–based research network,^14^ which included 32 primary care practices in the Philadelphia metropolitan area, including southeastern Pennsylvania and southern New Jersey. Data were extracted from CHOP’s electronic health record for all scheduled in-person preventive care visits among patients aged younger than 19 years in the CHOP primary care network between January 1, 2009, and December 31, 2023. The primary hypothesis and analysis plan were established prior to data collection. This study followed the Strengthening of the Reporting of Observational Studies in Epidemiology (STROBE) reporting guideline. The CHOP institutional review board determined this study to be exempt, and granted a waiver of informed consent, because the study was a minimal-risk secondary analysis of electronic health record data that could not practicably be carried out without a waiver.

### Measures

#### Outcome

Our outcome of interest was the daily rate of missed preventive care visits. We classified scheduled preventive care visits as missed (no-shows or same-day cancellations) vs all other visit statuses (eg, attended, left without being seen, or canceled or rescheduled prior to appointment date). We then calculated the total daily number of missed preventive care visits and the total daily number of scheduled preventive care visits at each primary care practice.

#### Exposure

Our primary exposure was maximum daily temperature (in degrees Fahrenheit). Daily temperature data were downloaded from Climate Data Online, provided by the National Oceanic and Atmospheric Administration’s National Centers for Environmental Information. We used the Global Historical Climate Network daily (GHCNd) dataset,^15^ which includes observations from monitoring stations throughout the US. We linked daily maximum temperature values to each primary care practice on each day of the study period based on the mean value for all monitoring stations within the same county as the practice. We also linked daily rainfall and snowfall data from the GHCNd using the same approach.

#### Covariates

Patient-level covariates, included for descriptive purposes and stratified analyses, were extracted from the electronic health record and included age at visit (categorized as <2, 2-5, 6-12, or 13-18 years), sex, race and ethnicity, and insurance type—public (Medicaid or Children’s Health Insurance Program [CHIP]), commercial, or other (included payers classified in the electronic health record as charity care, other, or unassigned). Race and ethnicity were ascertained according to the data available in the electronic health record and were included in the analysis for descriptive purposes as a marker of exposure to structural racism, not as biological variables. Categories were Asian, Hispanic or Latino, non-Hispanic Black (hereafter, *Black*), non-Hispanic White (hereafter, *White*), and other (included American Indian or Alaska Native, Native Hawaiian or Other Pacific Islander, multiracial, or recorded as “other” in the electronic health record); categories denoted “choose not to disclose,” “unknown,” or “missing” were classified as missing.

Census tract–level Child Opportunity Index (COI) 2.0^16^ scores from 2015 were linked to patients based on their geocoded home address as a multidimensional measure of neighborhood environmental resources and risks. We focused on the social and economic domain score, as the overall index includes a measure of temperature (number of summer days with temperatures above 90 °F) in the summary score. We used standard COI categories to classify patients as having neighborhood socioeconomic levels that were very low, low, moderate, high, or very high, defined based on quintiles of COI scores across all census tracts in the US.

### Statistical Analysis

We used generalized linear quasi-Poisson models to estimate associations of daily maximum temperatures with rate of missed primary care preventive visits. The daily count of missed visits was modeled as the dependent variable, with the log of the daily number of scheduled well visits as an offset. In our primary analysis, we did not include lagged temperature values, as we hypothesized that the temperature on the day of the visit was most relevant to no-show or same-day visit cancellations.^11^ Maximum daily temperature was modeled using a natural cubic spline term with 5 degrees of freedom to account for nonlinearity in the association with missed preventive care visits. Optimal number of knots was chosen by comparing the quasi-Akaike information criterion values for models with 1 to 6 degrees of freedom. We additionally included fixed effects for month and year to account for secular trends and seasonality, a fixed effect for practice to account for differences in missed visits across primary care practices, and the following day-level variables: day of the week and holiday status, rainfall (categorized as <75th percentile, 75th-95th percentile, or >95th percentile), and snowfall (binary; any vs none). In a secondary analysis, we examined associations of rainfall and snowfall with the rate of missed visits.

Using the model, we estimated rate ratios (RRs) and 95% CIs for the association of maximum daily temperatures with rate of missed preventive care visits, comparing values against the overall median temperature across the full temperature distribution in the study period (67.3 °F). Following prior work,^11^ we report estimates for maximum daily temperatures at the 5th, 10th, 25th, 75th, 90th, and 95th percentiles of the temperature distribution to demonstrate associations across a wide range of temperatures.

As in a prior study among Philadelphia adults,^11^ we observed linear trends in the association between temperature and missed preventive care visits above approximately the 90th percentile (88.0 °F) and below the 10th percentile (41.5 °F). As such, we conducted additional models with temperature modeled as a piecewise linear variable to estimate the relative risk of missed visits for each additional 1 °F above or below a threshold. This approach, which facilitates interpretation of results, has been used in prior studies of the association between temperature and health outcomes.^11,17,18^ For this analysis, we stratified into warm months (May-October) and cold months (November-April). In warm months, we calculated the temperature variable as maximum daily temperature minus 88.0 °F to estimate the relative risk for each 1 °F above the 90th percentile of the temperature distribution. Temperature values below 88.0 °F were coded as 0. In cold months, we calculated the temperature variable as 41.5 °F minus the maximum daily temperature to estimate the relative risk for each 1 °F below the 10th percentile. Temperatures above 41.5 °F were coded as 0. Models were adjusted as described previously, except that we did not adjust for snowfall in the warm month models.

We examined models stratified according to patient age (<2, 2-5, 6-12, or 13-18 years), insurance type (commercial, Medicaid or CHIP, or other), and COI neighborhood social and economic domain categories (very low, low, medium, high, or very high), based on a priori hypotheses that associations of temperature with missed primary care visits might differ according to age and SES. The Cochran *Q* statistic was used to test for heterogeneity in associations of temperature with rate of missed visits across strata of insurance type or COI category.^19^

We conducted several sensitivity analyses. First, we modeled the rate of missed visits as a count rather than a rate. Second, we considered a simpler model with 3 degrees of freedom in the association of temperature with rate of missed visits. Third, we adjusted for daily air pollution (particulate matter with diameter of 2.5 µm [PM_2.5_]) using the US Environmental Protection Agency’s daily Air Quality System data.^20^ Fourth, we examined no-show and same-day cancellations separately. Fifth, as individual patients were included multiple times in the daily time series and might have multiple missed visits, we stratified by whether patients had missed a visit in the 2 years prior to each appointment date to assess whether associations differed between patients with and without a history of missing preventive care visits. Sixth, we examined the minimum daily temperature instead of maximum daily temperature. Seventh, we considered lags of up to 2 days to account for possible short-term lagged associations of extreme temperatures with visit attendance on subsequent days. Eighth, we stratified by calendar year to assess whether the association of temperatures and missed visits varied over the study period.

We used the DLNM package^21^ in RStudio, version 2024.12.1 + 563 (RStudio, PBC), to implement the statistical models, extract RRs and 95% CIs, and produce plots. We used 2-sided *P* < .05 to indicate statistical significance for all tests. Analyses were conducted between March and October 2025.

## Results

### Study Population and Visit Characteristics

Our analysis included 504 428 unique children with a scheduled preventive care visit appointment in the CHOP primary care network between 2009 and 2023, of whom 49% were female and 51% were male; less than 1% had unidentified sex. In all, 5% of patients identified as Asian, 30% as Black, 8% as Hispanic or Latino, 44% as White, and 12% as other race and ethnicity; 1% had missing race and ethnicity data. Mean (SD) age was 6.0 (5.7) years. A total of 36% were insured by Medicaid or CHIP, and 27% resided in very low SES neighborhoods (Table 1). Of the 4 137 542 scheduled preventive care visits, 2 979 010 (72%) were attended, 533 404 (13%) were missed, and 625 128 (15%) were canceled prior to the appointment date or resulted in the patient leaving without being seen. The percentage of missed visits was uniform among age groups and sexes but varied across other demographic subgroups. Rates of missed visits were highest among Black patients (22%); patients with insurance other than commercial payers, Medicaid, or CHIP (36%); and patients from very low SES neighborhoods (22%) (Table 2).

**Table 1.** Characteristics of Patients With a Scheduled Preventive Care Visit, 2009 to 2023

Characteristic	Patients, No. (%) (N = 504 428)
Sex	
Female	246 982 (49)
Male	257 429 (51)
Unidentified	17 (<1)
Race and ethnicity	
Asian	24 335 (5)
Hispanic or Latino	42 413 (8)
Non-Hispanic Black	153 003 (30)
Non-Hispanic White	222 722 (44)
Other^a^	58 034 (12)
Missing^b^	3921 (1)
Payer	
Commercial	289 299 (57)
Medicaid or CHIP	180 738 (36)
Other^c^	26 502 (5)
Missing	7889 (2)
Neighborhood SES^d^	
Very low	135 092 (27)
Low	49 376 (10)
Moderate	62 495 (12)
High	89 246 (18)
Very high	158 877 (32)
Missing	9342 (2)

Abbreviations: CHIP, Children’s Health Insurance Program; SES, socioeconomic status.

^a^ Other race and ethnicity included American Indian or Alaska Native, multiracial, Native Hawaiian or Other Pacific Islander, or recorded as “other” in the electronic health record.

^b^ Categories denoted “choose not to disclose,” “unknown,” or “missing” were categorized as missing.

^c^ Included payers classified in the electronic health record as charity care, other, or unassigned.

^d^ Determined based on quintiles of the Child Opportunity Index social and economic domain score.

**Table 2.** Visit Status by Demographic Characteristics of Patients With a Scheduled Preventive Care Visit, 2009 to 2023

Characteristic	Scheduled appointments			
	Total No.	No. (row %)		
		Attended	Missed	Other^a^
Total appointments, No.	4 137 542	2 979 010 (72)	533 404 (13)	625 128 (15)
Age group, y				
<2	1 537 427	1 184 565 (77)	169 127 (11)	183 735 (12)
2-5	838 655	587 666 (70)	119 723 (14)	131 266 (16)
6-12	916 504	635 897 (69)	127 482 (14)	153 125 (17)
13-18	844 956	570 882 (68)	117 072 (14)	157 002 (19)
Sex				
Female	2 022 019	1 453 886 (72)	260 569 (13)	307 564 (15)
Male	2 115 393	1 525 019 (72)	272 824 (13)	317 550 (15)
Unidentified	130	105 (81)	11 (8)	14 (11)
Race and ethnicity				
Asian	172 698	130 798 (76)	10 856 (6)	31 044 (18)
Hispanic or Latino	342 379	246 967 (72)	45 054 (13)	50 358 (15)
Non-Hispanic Black	1 467 958	977 848 (67)	319 997 (22)	170 113 (12)
Non-Hispanic White	1 700 406	1 293 031 (76)	106 707 (6)	300 668 (18)
Other^b^	430 186	313 171 (73)	47 788 (11)	69 227 (16)
Missing^c^	23 915	17 195 (72)	3002 (13)	3718 (16)
Payer				
Commercial	2 220 912	1 766 889 (80)	128 862 (6)	325 161 (15)
Medicaid or CHIP	1 676 898	1 153 539 (69)	327 214 (20)	196 145 (12)
Other^d^	212 216	32 304 (15)	76 830 (36)	103 082 (49)
Missing	27 516	26 278 (96)	498 (2)	740 (3)
Neighborhood SES^e^				
Very low	1 333 708	894 872 (67)	294 297 (22)	144 539 (11)
Low	367 585	262 151 (71)	52 393 (14)	53 041 (14)
Moderate	496 112	365 418 (74)	52 504 (11)	78 190 (16)
High	687 620	512 335 (75)	56 195 (8)	119 090 (17)
Very high	1 196 000	904 788 (76)	69 543 (6)	221 669 (19)
Missing	56 517	39 446 (70)	8472 (15)	8599 (15)

Abbreviations: CHIP, Children’s Health Insurance Program; SES, socioeconomic status.

^a^ Includes scheduled appointments that were canceled (not on the same day) or resulted in the patient leaving without being seen, not resulting in a completed visit.

^b^ Other race and ethnicity included American Indian or Alaska Native, multiracial, Native Hawaiian or Other Pacific Islander, or recorded as “other” in the electronic health record.

^c^ Categories denoted “choose not to disclose,” “unknown,” or “missing” were categorized as missing.

^d^ Included payers classified in the electronic health record as charity care, other, or unassigned.

^e^ Determined based on quintiles of the Child Opportunity Index social and economic domain score.

### Temperature, Rainfall, and Snowfall Distribution

From May through October (warm months), the maximum daily temperature ranged from 42.8 °F to 104.5 °F, with a median of 80.9 °F. From November through April (cold months), the maximum daily temperature ranged from 13.1 °F to 92.7 °F, with a median of 51.1 °F. Throughout the study period, daily rainfall ranged from 0 to 15.5 cm, with a median of 0.3 cm, and daily snowfall ranged from 0 to 35.6 cm, with a median of 0.0 cm (eTable 1 in Supplement 1).

### Associations of Daily Maximum Temperature With Rates of Missed Visits

The fully adjusted generalized linear quasi-Poisson model showed that rates of missed visits increased as maximum daily temperature decreased below 41.5 °F and increased above 88.0 °F (Figure). At 36.5 °F (5th percentile) and 91.0 °F (95th percentile), the RRs for missed visits were 1.05 (95% CI, 1.04-1.07) and 1.04 (95% CI, 1.03-1.06), respectively, compared with the rate at the median temperature of 67.3 °F (Table 3). There was also evidence of linear associations between maximum daily temperature and the rate of missed visits at the extremes of maximum daily temperature (Figure and Table 4). During cold months, each 1 °F decrease below 41.5 °F was associated with a higher rate of missed visits (RR, 1.01; 95% CI, 1.01-1.01). During warm months, each 1 °F increase above 88.0 °F was also associated with a higher rate of missed visits (RR, 1.01; 95% CI, 1.00-1.01) (Table 4).

**Figure.**
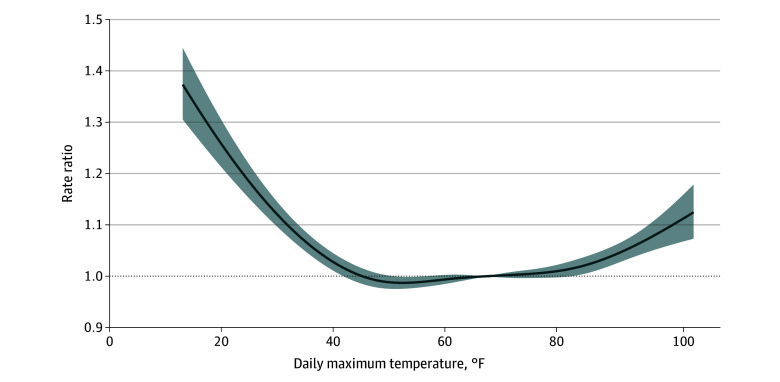
Line Graph of Daily Maximum Temperature vs Rate Ratio for Missed Pediatric Preventive Care Visits, 2009 to 2023 Estimated using a generalized linear quasi-Poisson distributed model, with the natural log of the total number of daily scheduled visits as an offset term. Daily maximum temperature was modeled using natural cubic splines to capture nonlinear associations, adjusted for day of the week, month, year, daily rainfall, daily snowfall, and practice fixed effects. Estimated rate ratios are in reference to the median temperature (67.34 °F).

**Table 3.** RR Estimates Between Different Percentiles of Daily Maximum Temperature and Rates of Missed Pediatric Preventive Care Visits

Maximum daily temperature percentile	Temperature, °F	RR (95% CI)
5th	36.5	1.05 (1.04-1.07)
10th	41.5	1.02 (1.00-1.04)
25th	51.5	0.99 (0.98-1.00)
50th	67.3	1.00 [Reference]
75th	81.3	1.01 (1.00-1.03)
90th	88.0	1.03 (1.01-1.05)
95th	91.0	1.04 (1.03-1.06)

Abbreviation: RR, rate ratio.

**Table 4.** RRs of Missed Preventive Care Visits by Patient Age, Payer Group, and Neighborhood SES Across Cold and Warm Months^a^

Characteristic	Months			
	Cold		Warm	
	RR (95% CI)^b^	*P* value^c^	RR (95% CI)^b^	*P* value^c^
Overall	1.01 (1.01-1.01)	NA	1.01 (1.00-1.01)	NA
Age group, y				
<2	1.02 (1.01-1.02)	<.001	1.01 (1.00-1.01)	.95
2-5	1.01 (1.01-1.01)		1.01 (1.00-1.01)	
6-12	1.01 (1.01-1.02)		1.01 (1.00-1.01)	
13-18	1.01 (1.01-1.01)		1.01 (1.00-1.01)	
Payer group				
Commercial	1.02 (1.02-1.02)	<.001	1.00 (1.00-1.01)	.06
Medicaid or CHIP	1.01 (1.01-1.01)		1.01 (1.00-1.01)	
Other^d^	1.00 (1.00-1.01)		1.00 (0.99-1.00)	
Neighborhood SES^e^				
Very low	1.01 (1.01-1.01)	<.001	1.01 (1.00-1.01)	.009
Low	1.01 (1.01-1.01)		1.00 (0.99-1.00)	
Moderate	1.01 (1.01-1.02)		1.00 (1.00-1.01)	
High	1.02 (1.01-1.02)		1.01 (1.00-1.02)	
Very high	1.02 (1.02-1.03)		1.00 (0.99-1.01)	

Abbreviations: CHIP, Children’s Health Insurance Program; NA, not applicable; RR, rate ratio; SES, socioeconomic status.

^a^ Based on observed linear associations above the 90th percentile of maximum daily temperature (88.0 °F) in warm months (May-October) and below the 10th percentile of maximum daily temperature (41.5 °F) in cold months (November-April), we modeled temperature with piecewise linear terms to estimate associations for each 1 °F increase in temperature above 88.0 °F in warm months and for each 1 °F decrease in temperature below 41.5 °F in cold months.

^b^ Models were adjusted for day of the week, month, year, rainfall, snowfall, and practice fixed effects. Models were stratified by Child Opportunity Index social and economic category, age, and payer.

^c^ The Cochran *Q* test statistic was used to test for heterogeneity between subgroups.

^d^ Included payers classified in the electronic health record as charity care, other, or unassigned.

^e^ Determined based on quintiles of the Child Opportunity Index Social and Economic Domain score.

Significant heterogeneity across patient age, insurance payer, and neighborhood SES subgroups was observed consistently during cold months but not during warm months (Table 4). During cold months, colder temperatures were associated with a slightly higher rate of missed appointments among children aged less than 2 years compared with older children; children with commercial insurance compared with Medicaid, CHIP, or other payers (RR, 1.02; 95% CI, 1.02-1.02); and children from high or very high SES neighborhoods (Table 4). During the warm months, associations were largely homogenous across categories of age and insurance payer, but the rate was higher among children in the very low and the high neighborhood SES categories vs low, moderate, and very high SES (Table 4). Nonlinear stratified results are presented in eFigure 1 and eTable 2 in Supplement 1.

eTable 3 in Supplement 1 shows the associations of daily rainfall and snowfall with rate of missed visits. Compared with days with rainfall below the 75th percentile, days with rainfall between the 75th and 90th percentiles had a higher rate of missed visits (RR, 1.04; 95% CI, 1.03-1.05), as did days with rainfall above the 90th percentile (RR, 1.08; 95% CI, 1.07-1.09). Rates of missed visits associated with rainfall were higher during cold months compared with warm months. Days with any snowfall had a higher rate of missed visits overall (RR, 1.33; 95% CI, 1.31-1.35) compared with days without snow.

### Sensitivity Analyses

In sensitivity analyses that examined missed visits as a count rather than a rate, examined a simpler model with 3 degrees of freedom, adjusted for daily PM_2.5_ concentration, and examined minimum rather than maximum daily temperature, results were similar to the primary analysis (eFigure 2 and eTable 4 in Supplement 1). Daily PM_2.5_ concentration in the very unhealthy vs healthy category was associated with a higher rate of missed visits (RR, 1.19; 95% CI, 1.05-1.35), but other PM_2.5_ categories were not associated with higher rates of missed visits. In models that separated no-shows from same-day cancellations, cold temperatures were associated with a higher rate of same-day cancellations, while both cold and hot temperatures were associated with a higher rate of no-shows (eFigure 2 and eTable 4 in Supplement 1). When we stratified by whether patients had a history of missed visits, we found that extreme heat was associated with a slightly higher rate of missed visits for patients with a prior missed visit and extreme cold was associated with a higher rate for patients without a prior missed visit. When we considered lags of up to 2 days, we found the highest rates of missed visits when extreme temperatures were on the same day of the visit and slightly higher cumulative rates when extreme temperatures occurred over the 2 days before the visit (eFigure 3 and eTable 5 in Supplement 1). In models that stratified by year, the association between extreme temperatures and missed visits was consistent overall across periods (eFigure 4 and eTable 6 in Supplement 1).

## Discussion

In this time series analysis of preventive care visits in a large pediatric primary care network over a 15-year period, extreme temperatures (both hot and cold) were associated with a higher rate of missed pediatric preventive care visits after adjustment for daily covariates, secular trends, and practice fixed effects. Rates of missed visits were somewhat higher in colder compared with warmer months, and there was evidence of heterogeneity in associations by age, insurance type, and neighborhood SES. Missed primary care visits might delay children’s receipt of needed preventive care such as vaccinations and developmental screening and have been found to increase risk of ambulatory care–sensitive hospitalizations.^7^ Missed visits also negatively impact primary care practice workflows and increase costs.^6,8^

Prior studies in pediatric populations have demonstrated that extreme temperatures, especially extreme heat, are associated with higher rates of emergency department visits among children. For example, an analysis of claims data from insured US children found a 30% higher rate of emergency department visits for heat-related illnesses on days at the 95th percentile of county-level heat distribution relative to the median.^5^ Similarly, 2 studies in New York City found excess risk of emergency department visits^3,4^ and hospitalizations^3^ associated with higher ambient temperatures in warm months, although associations differed by age group. However, associations of extreme temperature with primary care visits have not been comprehensively studied in children. Fitzpatrick et al^11^ examined associations between maximum daily temperatures and missed primary care visits among adults in 13 large, university outpatient primary care practices in Philadelphia and found the rate of missed visits to increase by 0.72% per degree decrease below 39 °F and by 0.64% per degree increase above 89 °F, similar in magnitude to our findings among children in the same region.

Missed pediatric preventive care visits have multiple causes—for example, survey data from parents at urban primary care practices identified transportation problems, difficulty getting time off work, and forgetting appointments as common barriers.^9,10^ Sociodemographic differences in the rate of missed primary care visits have been reported in prior studies, with older children,^9^ children with public insurance,^13^ and children with food insecurity or other unmet social needs^10,12^ found to have higher rates. Consistent with prior findings, the percentage of missed visits was higher in our study population among non–commercially insured children and children from low SES neighborhoods and was lower among children aged younger than 2 years compared with older children. We hypothesized that extreme temperatures would be associated with higher rates of missed preventive care visits among publicly insured patients and those from low SES neighborhoods, potentially due to greater transportation challenges that might be impacted by weather. However, we found the opposite outcome in cold months, with commercially insured children and those living in higher-opportunity neighborhoods having the highest rates of missed visits in decreasing temperatures. It may be that the higher baseline rates of missed visits among non–commercially insured children and children from very low SES neighborhoods are driven by other factors and are less sensitive to impacts of temperature, whereas associations with temperature were more apparent among the demographic subgroups with lower baseline rates of missed visits (ie, commercially insured children and children from high SES neighborhoods). Alternatively, families with higher SES might have more flexibility to reschedule appointments, while lower SES families might need to attend regardless of extreme temperatures due to limited appointment availability or challenges in getting time off from work. Future qualitative research is needed to better understand what underlies these differences.

To mitigate potential impacts of extreme temperatures on primary care attendance, primary care practices might consider testing strategies such as expanding telemedicine when extreme temperatures are expected. Telehealth appointments have been associated with reduced likelihood of no-shows at primary care or internal medicine appointments among children^22,23^ and adults,^24,25,26^ although not all components of preventive care can be completed via telehealth (eg, vaccinations). Future work is needed to assess the effect of converting missed visits to telehealth visits in the context of extreme temperatures. Additional approaches such as facilitating transportation for patients with transportation challenges (eg, by partnering with rideshare vendors) could be explored. Practices could also implement weather-monitoring systems that trigger proactive outreach to patients with scheduled appointments when extreme temperatures are forecasted, offering flexible rescheduling options. In addition, flexible scheduling policies, such as relaxed same-day cancellation penalties during extreme weather events or extended appointment availability in the days following extreme temperature events, may help patients maintain access to necessary preventive care. While our findings suggest temperature-related barriers to care access exist, practices should weigh the costs and feasibility of interventions against their local climate patterns and patient population needs. Further research is needed to evaluate the effectiveness and cost-effectiveness of these potential strategies.

### Strengths and Limitations

Strengths of this study include the large sample of children and adolescents in a primary care population, the socioeconomic diversity of the study population, and the long follow-up period. The CHOP primary care network includes children in urban, suburban, and semirural settings with 32 practices across 2 states. However, generalizability was limited by including only participants in 1 geographic region. Also, because we aggregated daily missed visits and temperatures at the primary care practice level, our temperature exposure does not reflect fine-grained differences that may be experienced among individuals in different neighborhoods (eg, due to the urban heat island effect) and may lead to exposure misclassification. Future work linking higher spatial resolution (eg, census tract–level) temperature data to individual patient addresses is needed to confirm these findings. In addition, we lacked data on individual-level characteristics that might also impact missed preventive care, such as vehicle access. We were not able to reliably determine the reason for each missed visit, as such information is not recorded consistently across the network. This limited our ability to examine visits that were canceled or rescheduled prior to each visit date, although it is possible that such cancellations could have occurred due to weather forecasts.

## Conclusions

This cross-sectional study found that extreme temperatures were associated with higher rates of missed pediatric primary care visits. As climate scientists expect an increasing frequency and intensity of extreme temperatures, hospital systems and clinics may anticipate temperature-related impacts of health care and consider mitigating solutions.

## Notes

Supplement 1. **eTable 1.** Temperature, Precipitation, and Snowfall Distribution Across Cold and Warm Seasons, 2009-2023 **eFigure 1.** Daily Maximum Temperature vs Rate Ratio for Missed Pediatric Preventive Visits, 2009-2023, by Patient Age, Payer Group, and Neighborhood Socioeconomic Status **eTable 2.** Rate Ratio and 95% CI Estimates for Different Percentiles of Daily Maximum Temperature (Tmax) and Rates of Missed Pediatric Preventive Visits, by Patient Age, Payer Group, and Neighborhood Socioeconomic Status **eTable 3.** Rate Ratio and 95% CI Estimates for the Association Between Daily Rainfall, Snowfall, and Rates of Missed Pediatric Preventive Visits **eFigure 2.** Sensitivity Analyses: Rate Ratios and 95% CIs for Associations Between Daily Temperatures and Missed Pediatric Preventive Visits **eTable 4.** Sensitivity Analyses: Rate Ratios and 95% CIs for Associations Between Different Percentiles of Daily Temperatures and Missed Pediatric Preventive Visits **eFigure 3.** Rate Ratios and 95% CIs for Associations Between Daily Maximum Temperature (Tmax) and Missed Pediatric Preventive Visits Across Lags of 0 to 2 Days **eTable 5.** Cumulative Rate Ratios and 95% CIs for Difference Percentiles of Daily Maximum Temperature and Missed Pediatric Preventive Visits Over a 2-Day Lag Period **eFigure 4.** Rate Ratios and 95% CIs for Associations Between Daily Maximum Temperature (Tmax) and Missed Pediatric Preventive Visits Over 3-Year Intervals, 2009-2023 **eTable 6.** Rate Ratios and 95% CIs for Associations Between Different Percentiles of Daily Temperatures and Missed Pediatric Preventive Visits Over 3-Year Intervals, 2009-2023

Supplement 2.
Data Sharing Statement
