# Supplementary material for: Extreme Temperatures and Missed Pediatric Preventive Care Visits
Source: JAMA Netw Open. 2026 Apr 27;9(4):e2610114. doi: 10.1001/jamanetworkopen.2026.10114 (PMC13122397; doi:10.1001/jamanetworkopen.2026.10114)
Supplement: Supplement 1. — eTable 1. Temperature, Precipitation, and Snowfall Distribution Across Cold and Warm Seasons, 2009-2023 eFigure 1. Daily Maximum Temperature vs Rate Ratio for Missed Pediatric Preventive Visits, 2009-2023, by Patient Age, Payer Group, and Neighborhood Socioeconomic Status eTable 2. Rate Ratio and 95% CI Estimates for Different Percentiles of Daily Maximum Temperature (Tmax) and Rates of Missed Pediatric Preventive Visits, by Patient Age, Payer Group, and Neighborhood Socioeconomic Status eTable 3. Rate Ratio and 95% CI Estimates for the Association Between Daily Rainfall, Snowfall, and Rates of Missed Pediatric Preventive Visits eFigure 2. Sensitivity Analyses: Rate Ratios and 95% CIs for Associations Between Daily Temperatures and Missed Pediatric Preventive Visits eTable 4. Sensitivity Analyses: Rate Ratios and 95% CIs for Associations Between Different Percentiles of Daily Temperatures and Missed Pediatric Preventive Visits eFigure 3. Rate Ratios and 95% CIs for Associations Between Daily Maximum Temperature (Tmax) and Missed Pediatric Preventive Visits Across Lags of 0 to 2 Days eTable 5. Cumulative Rate Ratios and 95% CIs for Difference Percentiles of Daily Maximum Temperature and Missed Pediatric Preventive Visits Over a 2-Day Lag Period eFigure 4. Rate Ratios and 95% CIs for Associations Between Daily Maximum Temperature (Tmax) and Missed Pediatric Preventive Visits Over 3-Year Intervals, 2009-2023 eTable 6. Rate Ratios and 95% CIs for Associations Between Different Percentiles of Daily Temperatures and Missed Pediatric Preventive Visits Over 3-Year Intervals, 2009-2023 [file jamanetwopen-e2610114-s001.pdf]

Supplementary Online Content

Mayne SL, Ramachandran J, Sharma P, et al. Extreme temperatures and missed pediatric preventive care visits. *JAMA Netw Open*. 2026;9(4):e2610114. doi:10.1001/jamanetworkopen.2026.10114

eTable 1. Temperature, Precipitation, and Snowfall Distribution Across Cold and Warm Seasons, 2009-2023

eFigure 1. Daily Maximum Temperature vs Rate Ratio for Missed Pediatric Preventive Visits, 2009-2023, by Patient Age, Payer Group, and Neighborhood Socioeconomic Status

eTable 2. Rate Ratio and 95% CI Estimates for Different Percentiles of Daily Maximum Temperature (Tmax) and Rates of Missed Pediatric Preventive Visits, by Patient Age, Payer Group, and Neighborhood Socioeconomic Status

eTable 3. Rate Ratio and 95% CI Estimates for the Association Between Daily Rainfall, Snowfall, and Rates of Missed Pediatric Preventive Visits

eFigure 2. Sensitivity Analyses: Rate Ratios and 95% CIs for Associations Between Daily Temperatures and Missed Pediatric Preventive Visits

eTable 4. Sensitivity Analyses: Rate Ratios and 95% CIs for Associations Between Different Percentiles of Daily Temperatures and Missed Pediatric Preventive Visits

eFigure 3. Rate Ratios and 95% CIs for Associations Between Daily Maximum Temperature (Tmax) and Missed Pediatric Preventive Visits Across Lags of 0 to 2 Days

eTable 5. Cumulative Rate Ratios and 95% CIs for Difference Percentiles of Daily Maximum Temperature and Missed Pediatric Preventive Visits Over a 2-Day Lag Period

eFigure 4. Rate Ratios and 95% CIs for Associations Between Daily Maximum Temperature (Tmax) and Missed Pediatric Preventive Visits Over 3-Year Intervals, 2009-2023

eTable 6. Rate Ratios and 95% CIs for Associations Between Different Percentiles of Daily Temperatures and Missed Pediatric Preventive Visits Over 3-Year Intervals, 2009-2023

This supplementary material has been provided by the authors to give readers additional information about their work.

© 2026 Mayne SL et al. *JAMA Network Open*.

eTable 1: Temperature, Precipitation, and Snowfall Distribution Across Cold and Warm Seasons, 2009-2023[1]

	Minimum	25th Percentile	Median	Mean	75th Percentile	Maximum
**Overall**						
Maximum Daily Temperature – Tmax (ºF)	13.10	51.53	67.34	65.90	81.26	104.54
Minimum Daily Temperature – Tmin (ºF)	-1.84	33.98	47.25	47.50	62.42	84.47
Daily Rainfall (in.)	0.00	0.00	0.01	0.14	0.11	6.07
Daily Snowfall (in.)	0.00	0.00	0.00	0.05	0.00	14.00
**Warm Months**						
Maximum Daily Temperature – Tmax (ºF)	42.80	73.04	80.90	79.33	86.54	104.54
Minimum Daily Temperature – Tmin (ºF)	25.04	53.51	62.06	60.49	68.45	84.47
Daily Rainfall (in.)	0.00	0.00	0.01	0.15	0.13	6.07
Daily Snowfall (in.)	0.00	0.00	0.00	0.00	0.00	0.00
**Cold Months**						
Maximum Daily Temperature – Tmax (ºF)	13.10	42.98	51.08	51.67	60.08	92.71
Minimum Daily Temperature – Tmin (ºF)	-1.84	27.08	33.53	33.74	40.64	69.02
Daily Rainfall (in.)	0.00	0.00	0.00	0.12	0.09	4.42
Daily Snowfall (in.)	0.00	0.00	0.00	0.11	0.00	14.00

[1]Cold months included November – April. Warm months included May – October.

© 2026 Mayne SL et al. *JAMA Network Open*.

eFigure 1: Daily Maximum Temperature vs Rate Ratio for Missed Pediatric Preventive Visits, 2009-2023 – by Patient Age, Payer Group, and Neighborhood Socioeconomic Status

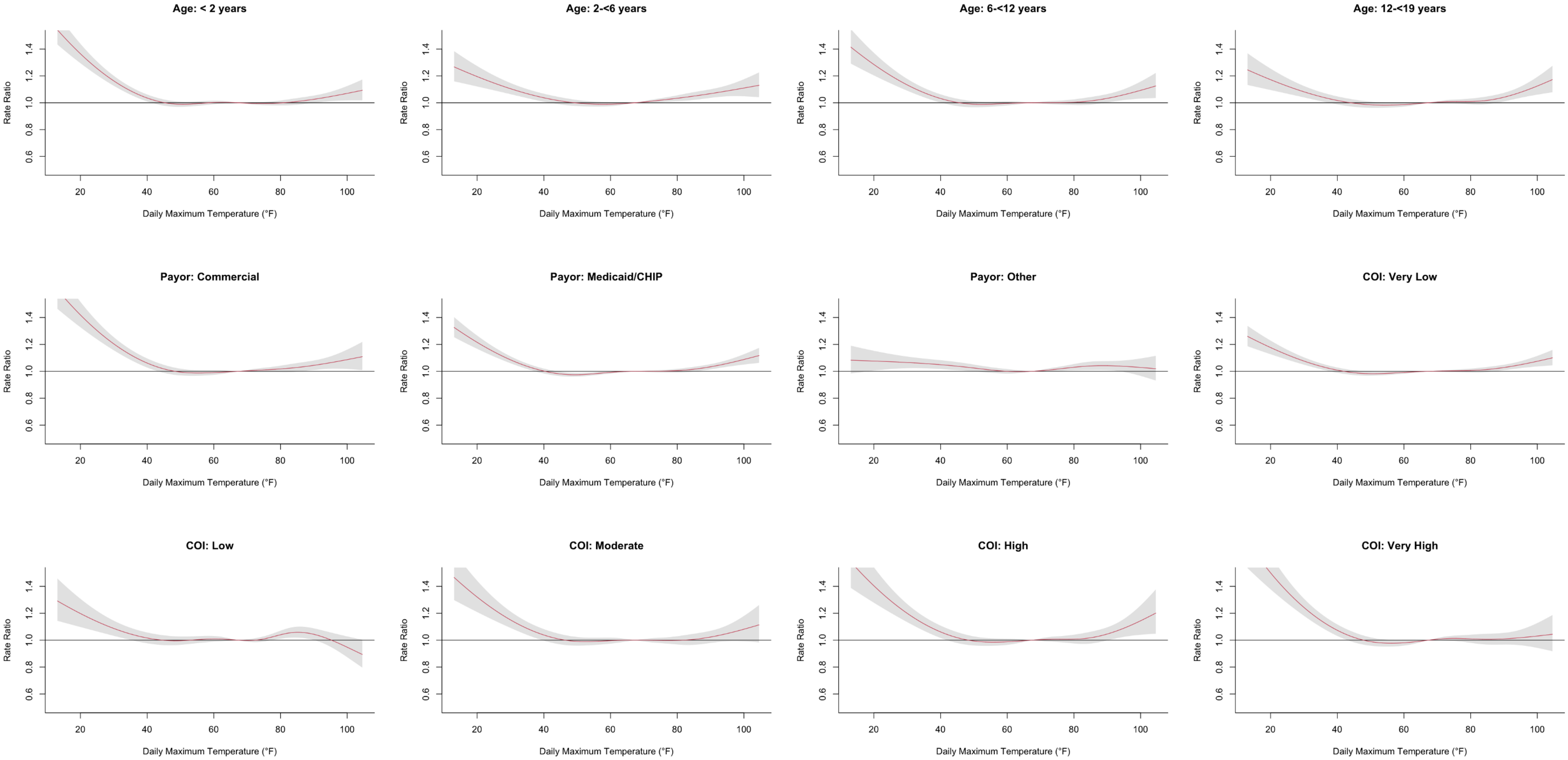

Estimated using separate generalized linear quasi-Poisson distributed models for each stratum, with the natural log of the total number of daily scheduled visits as an offset term. Daily maximum temperature Tmax was modeled using natural cubic splines to capture non-linear associations, adjusted for day of the week, month, year, daily precipitation, snowfall, and practice fixed effects. Estimated rate ratios are in reference to the median temperature (67.34°F**)**. COI indicates child opportunity index – social and economic domain as a proxy for neighborhood socioeconomic status.

© 2026 Mayne SL et al. *JAMA Network Open*.

eTable 2: Rate Ratio and 95% CI Estimates for Different Percentiles of Daily Maximum Temperature (Tmax) and Rates of Missed Pediatric Preventive Visits, by Patient Age, Payer Group, and Neighborhood Socioeconomic Status

	5th percentile	10th percentile	25th percentile	50th percentile	75th percentile	90th percentile	95th percentile
**Tmax (ºF)**	36.50	41.48	51.53	67.34	81.26	87.98	91.04
**Rate Ratio (95% CI)**							
**Age Group**							
<2 years	1.07 (1.04 – 1.10)	1.02 (1.00 – 1.05)	0.99 (0.97 – 1.01)	1.00 (Ref)	1.00 (0.98 – 1.02)	1.02 (0.99 – 1.05)	1.03 (1.00 – 1.06)
2 to <6 years	1.06 (1.03 – 1.09)	1.03 (1.00 – 1.06)	0.99 (0.97 – 1.02)	1.00 (Ref)	1.04 (1.01 – 1.06)	1.06 (1.03 – 1.09)	1.07 (1.04 – 1.11)
6 to <12 years	1.06 (1.03 – 1.09)	1.02 (0.99 – 1.05)	0.99 (0.97 – 1.01)	1.00 (Ref)	1.01 (0.98 – 1.03)	1.02 (0.99 – 1.05)	1.04 (1.01 – 1.07)
12 to <19 years	1.04 (1.00 – 1.07)	1.01 (0.98 – 1.04)	0.98 (0.96 – 1.00)	1.00 (Ref)	1.01 (0.99 – 1.03)	1.03 (1.00 – 1.06)	1.05 (1.02 – 1.08)
**Payor Group**							
Commercial	1.10 (1.07 – 1.14)	1.05 (1.01 – 1.08)	0.99 (0.97 – 1.01)	1.00 (Ref)	1.02 (0.99 – 1.05)	1.04 (1.00 – 1.07)	1.05 (1.01 – 1.09)
Medicaid/CHIP	1.03 (1.01 – 1.05)	0.99 (0.98 – 1.01)	0.98 (0.96 – 0.99)	1.00 (Ref)	1.01 (0.99 – 1.02)	1.03 (1.01 – 1.04)	1.04 (1.02 – 1.06)
Other	1.06 (1.02 – 1.09)	1.05 (1.01 – 1.08)	1.02 (1.00 – 1.04)	1.00 (Ref)	1.03 (1.01 – 1.06)	1.04 (1.01 – 1.08)	1.04 (1.01 – 1.08)
**Neighborhood Socioeconomic Status**							
Very Low	1.03 (1.01 – 1.05)	1.00 (0.98 – 1.02)	0.98 (0.97 – 1.00)	1.00 (Ref)	1.01 (0.99 – 1.02)	1.02 (1.00 – 1.04)	1.03 (1.01 – 1.05)
Low	1.04 (0.99 – 1.08)	1.01 (0.97 – 1.05)	1.00 (0.97 – 1.03)	1.00 (Ref)	1.05 (1.01 - 1.08)	1.05 (1.01 – 1.10)	1.04 (0.99 – 1.08)
Moderate	1.07 (1.03 – 1.12)	1.03 (0.99 – 1.07)	0.99 (0.96 – 1.02)	1.00 (Ref)	1.00 (0.97 – 1.03)	1.02 (0.97 – 1.06)	1.03 (0.98 – 1.08)
High	1.10 (1.05 – 1.15)	1.05 (1.00 – 1.09)	0.99 (0.96 – 1.02)	1.00 (Ref)	1.01 (0.97 – 1.05)	1.03 (0.99 – 1.08)	1.05 (1.00 – 1.11)
Very High	1.12 (1.08 – 1.17)	1.06 (1.02 – 1.10)	0.98 (0.96 – 1.01)	1.00 (Ref)	1.01 (0.98 – 1.04)	1.01 (0.97 – 1.05)	1.01 (0.97 – 1.06)

Neighborhood socioeconomic status was determined using the Child Opportunity Index Social and Economic domain score.

© 2026 Mayne SL et al. *JAMA Network Open*.

eTable3: Rate Ratio and 95% CI Estimates for the Association Between Daily Rainfall, Snowfall, and Rates of Missed Pediatric Preventive Visits[1]

	Rainfall <75th Percentile	Rainfall 75-90th Percentile	Rainfall > 90th Percentile	Snowfall 0 inches	Snowfall> 0 inches
	**RR**	**RR (95% CI)**	**RR (95% CI)**	**RR**	**RR (95% CI)**
Overall	Ref	1.04 (1.03, 1.05)	1.08 (1.07, 1.09)	Ref	1.33 (1.31, 1.35)
Cold Months	Ref	1.07 (1.05, 1.08)	1.11 (1.09, 1.13)	Ref	1.31 (1.28, 1.33)
Warm Months	Ref	1.02 (1.01, 1.03)	1.04 (1.03, 1.06)	NA	NA

[1]Rate ratios (RRs) estimated using a generalized linear quasi-Poisson distributed model, with the natural log of the total number of daily scheduled visits as an offset term. Models were adjusted for daily maximum temperature (modeled using natural cubic splines with 5 degrees of freedom), day of the week, month, year, and practice fixed effects.

© 2026 Mayne SL et al. *JAMA Network Open*.

eFigure 2: Sensitivity Analyses: Rate Ratios and 95% CIs for Associations between Daily Temperatures and Missed Pediatric Preventive Visits

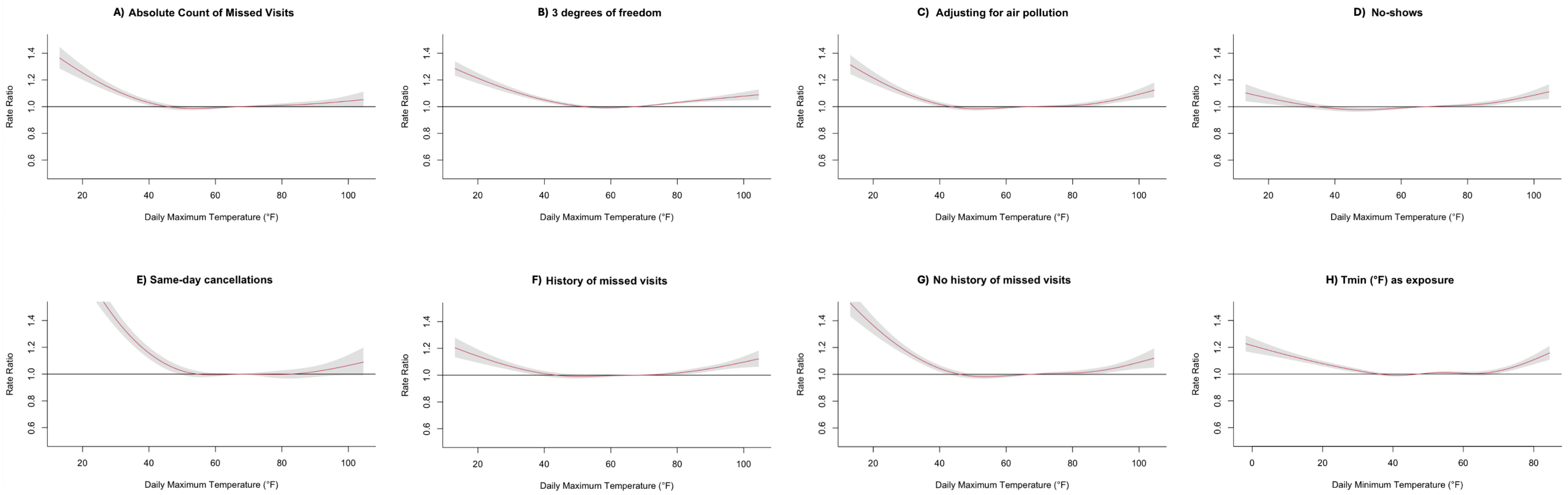

Estimated using generalized linear quasi-Poisson distributed models, with the natural log of the total number of daily scheduled visits as an offset term. Daily temperature was modeled using natural cubic splines to capture non-linear associations, adjusted for day of the week, month, year, daily precipitation, snowfall, and practice fixed effects. Figures present the following sensitivity analyses: A) modeling missed visits as a count rather than a rate by removing the offset term; B) including 3 rather than 5 degrees of freedom for Tmax; C) adjusting for daily particulate matter 2.5 (PM2.5) values; D) modeling no-shows as the outcome; E) modeling same-day cancellations as the outcome; F-G) stratifying by whether patients had a prior missed visits in the past two years (F) or not (G); and H) modeling daily minimum temperature (Tmin) as the exposure rather than Tmax.

© 2026 Mayne SL et al. *JAMA Network Open*.

eTable 4: Sensitivity Analyses: Rate Ratios and 95% CIs for Associations between Different Percentiles of Daily Temperatures and Missed Pediatric Preventive Visits

	5th percentile	10th percentile	25th percentile	50th percentile	75th percentile	90th percentile	95th percentile
Rate Ratio (95% CI)							
Tmax (ºF)	36.50	41.48	51.53	67.34	81.26	87.98	91.04
Main Model	1.05 (1.04 – 1.07)	1.02 (1.00 – 1.04)	0.99 (0.98 – 1.00)	1.00 (Ref)	1.01 (1.00 – 1.03)	1.03 (1.01 – 1.05)	1.04 (1.03 – 1.06)
Modeling Count of Missed Visits	1.06 (1.04 – 1.08)	1.02 (1.00 – 1.04)	0.99 (0.97 – 1.00)	1.00 (Ref)	1.01 (1.00 – 1.03)	1.02 (1.00 – 1.04)	1.02 (1.00 – 1.05)
Model with 3 degrees of freedom	1.07 (1.05 – 1.09)	1.04 (1.03 – 1.06)	1.00 (0.99 – 1.01)	1.00 (Ref)	1.03 (1.02 – 1.04)	1.05 (1.04 – 1.06)	1.06 (1.04 – 1.08)
Model adjusting for air pollution	1.04 (1.02 – 1.06)	1.01 (0.99 – 1.03)	0.98 (0.97 – 1.00)	1.00 (Ref)	1.01 (1.00 – 1.02)	1.03 (1.01 – 1.05)	1.04 (1.02 – 1.06)
Model with no-shows as outcome	0.99 (0.98 – 1.01)	0.98 (0.97 – 1.00)	0.98 (0.97 – 0.99)	1.00 (Ref)	1.01 (1.00 – 1.03)	1.03 (1.01 – 1.05)	1.04 (1.02 – 1.06)
Model with same-day cancellations as outcome	1.23 (1.18 – 1.28)	1.13 (1.09 – 1.17)	1.01 (0.99 – 1.04)	1.00 (Ref)	1.00 (0.97 – 1.03)	1.01 (0.97 – 1.05)	1.02 (0.98 – 1.07)
Model for patients with history of missed visits in preceding 2 years	1.03 (1.01 – 1.05)	1.01 (0.99 – 1.03)	0.99 (0.98 – 1.00)	1.00 (Ref)	1.02 (1.00 – 1.03)	1.04 (1.02 – 1.06)	1.05 (1.03 – 1.08)
Model for patients with no history of missed visits in preceding 2 years	1.08 (1.06 – 1.11)	1.03 (1.01– 1.05)	0.98 (0.97 – 1.00)	1.00 (Ref)	1.01 (0.99 – 1.03)	1.03 (1.00 – 1.05)	1.04 (1.01 – 1.06)
Tmin (ºF)	21.20	25.70	33.98	47.26	62.42	69.93	72.31
Main Model (Tmin as exposure)	1.07 (1.05 – 1.09)	1.05 (1.03 – 1.06)	1.01 (1.00 – 1.02)	1.00 (Ref)	1.00 (0.99 – 1.02)	1.02 (1.01 – 1.04)	1.04 (1.02 – 1.06)

Estimated using generalized linear quasi-Poisson distributed models, with the natural log of the total number of daily scheduled visits as an offset term. Daily temperature was modeled using natural cubic splines to capture non-linear associations, adjusted for day of the week, month, year, daily precipitation, snowfall, and practice fixed effects.

© 2026 Mayne SL et al. *JAMA Network Open*.

eFigure 3: Rate Ratios and 95% CIs for Associations Between Daily Maximum Temperature (Tmax) and Missed Pediatric Preventive Visits Across Lags of 0 to 2 Days

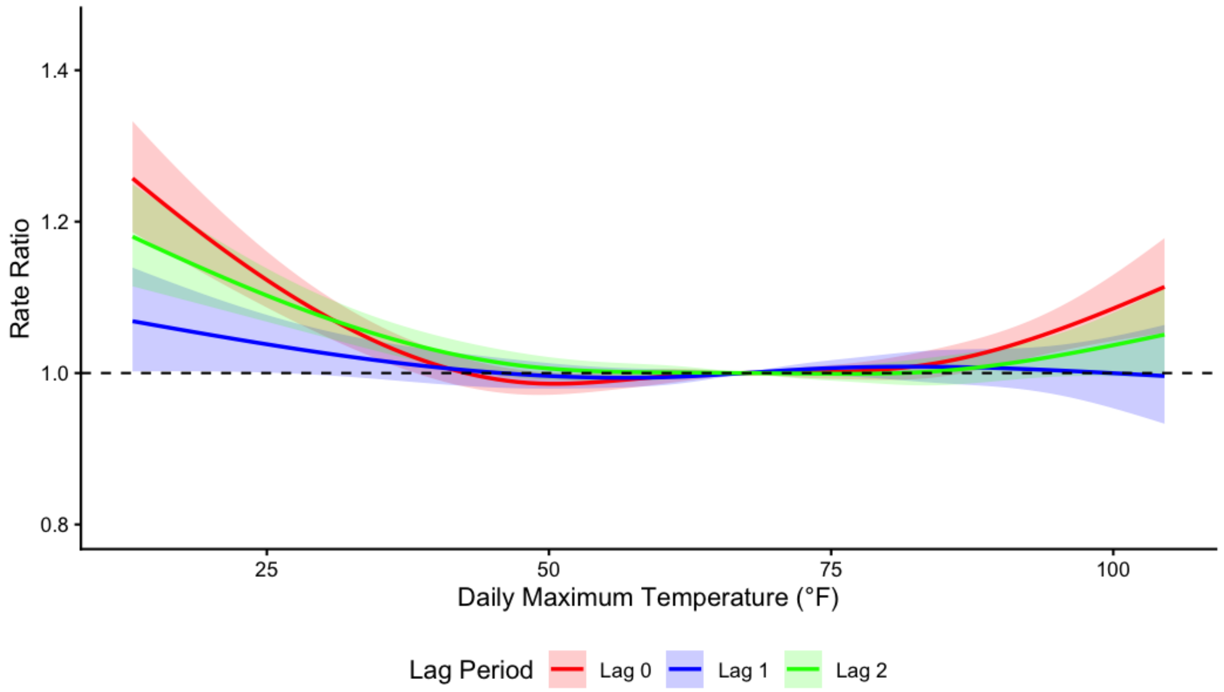

Estimated using generalized linear quasi-Poisson distributed models, with the natural log of the total number of daily scheduled visits as an offset term. Daily temperature was modeled using natural cubic splines to capture non-linear associations, adjusted for day of the week, month, year, daily precipitation, snowfall, and practice fixed effects. Models included lags of 0, 1, and 2 days.

© 2026 Mayne SL et al. *JAMA Network Open*.

eTable 5: Cumulative Rate Ratios and 95% CIs for Difference Percentiles of Daily Maximum Temperature and Missed Pediatric Preventive Visits Over a 2-Day Lag Period

Tmax Percentile	Temperature (°F)	Cumulative Rate Ratio (95% CI)
5th	36.50	1.09 (1.07 – 1.11)
10th	41.48	1.04 (1.01 – 1.06)
25th	51.53	0.99 (0.97 – 1.00)
50th	67.34	1.00 (Ref)
75th	81.26	1.02 (1.00 – 1.03)
90th	87.98	1.04 (1.02 – 1.06)
95th	91.04	1.06 (1.03 – 1.08)

Estimated using generalized linear quasi-Poisson distributed models, with the natural log of the total number of daily scheduled visits as an offset term. Daily temperature was modeled using natural cubic splines to capture non-linear associations, adjusted for day of the week, month, year, daily precipitation, snowfall, and practice fixed effects. Models included lags of 0, 1, and 2 days.

© 2026 Mayne SL et al. *JAMA Network Open*.

eFigure 4: Rate Ratios and 95% CIs for Associations Between Daily Maximum Temperature (Tmax) and Missed Pediatric Preventive Visits Over 3-Year Intervals, 2009-2023

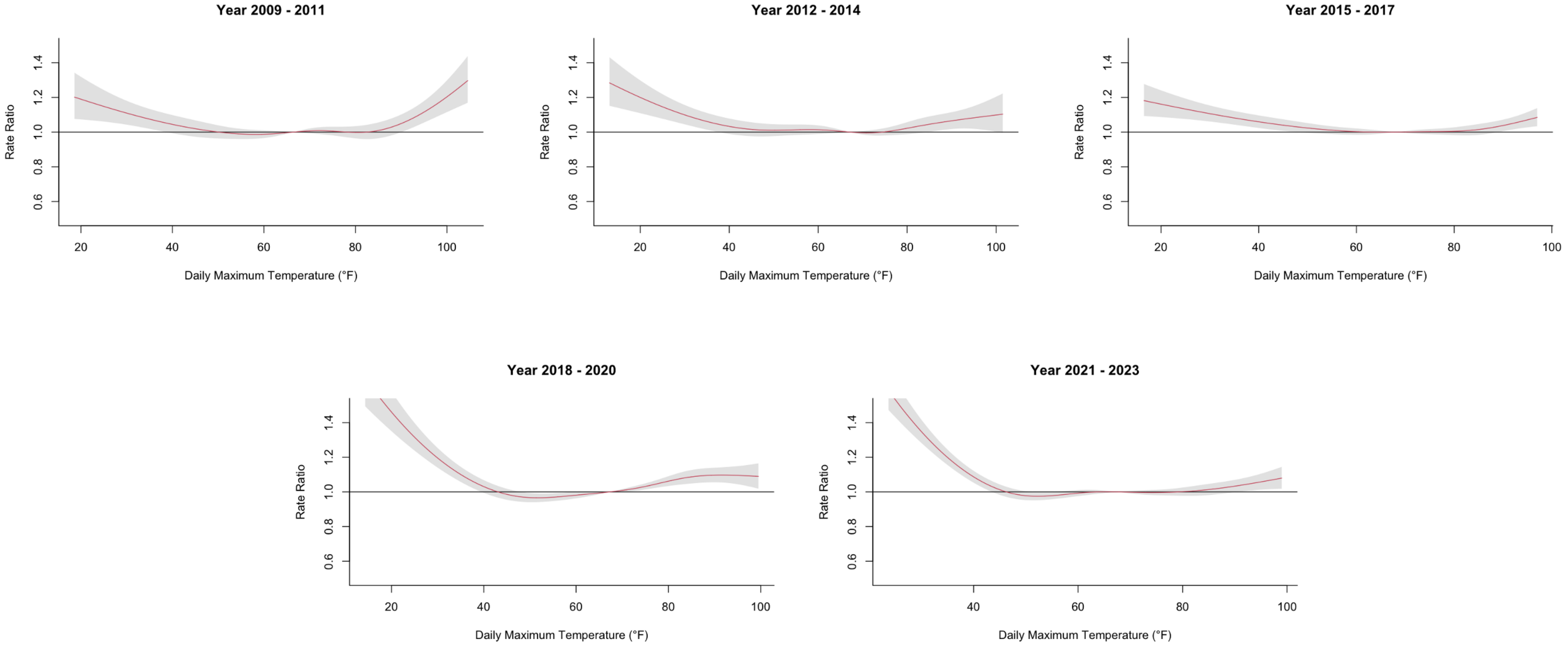

Estimated using generalized linear quasi-Poisson distributed models, with the natural log of the total number of daily scheduled visits as an offset term. Daily temperature was modeled using natural cubic splines to capture non-linear associations, adjusted for day of the week, month, year, daily precipitation, snowfall, and practice fixed effects. Data were stratified in 3-year increments to examine differences over the 15-year follow-up period.

© 2026 Mayne SL et al. *JAMA Network Open*.

eTable 6: Rate Ratios and 95% CIs for Associations between Different Percentiles of Daily Temperatures and Missed Pediatric Preventive Visits Over 3-Year Intervals, 2009-2023

Years	5th percentile	10th percentile	25th percentile	50th percentile	75th percentile	90th percentile	90th percentile
**2009 - 2011**							
Tmax (ºF)	34.52	37.99	51.56	66.47	80.96	87.98	91.04
Rate Ratio (95% CI)	1.08 (1.02 – 1.14)	1.06 (1.00 – 1.11)	1.00 (0.96 – 1.03)	1.00 (Ref)	1.00 (0.96 – 1.04)	1.03 (0.98 – 1.08)	1.06 (1.01 – 1.11)
**2012 - 2014**							
Tmax (ºF)	35.24	40.04	50.43	67.15	80.06	86.77	90.01
Rate Ratio (95% CI)	1.06 (1.01 – 1.11)	1.03 (0.99 – 1.08)	1.01 (0.98 – 1.04)	1.00 (Ref)	1.02 (0.99 – 1.06)	1.05 (1.01 – 1.10)	1.06 (1.02 – 1.11)
**2015 - 2017**							
Tmax (ºF)	35.06	41.54	53.24	68.00	82.40	88.52	91.04
Rate Ratio (95% CI)	1.08 (1.04 – 1.12)	1.05 (1.02 – 1.09)	1.01 (0.99 – 1.04)	1.00 (Ref)	1.01 (0.98 – 1.04)	1.03 (1.00 – 1.06)	1.04 (1.01 – 1
**2018 - 2020**							
Tmax (ºF)	37.34	41.45	50.45	67.28	82.04	88.58	91.07
Rate Ratio (95% CI)	1.06 (1.03 – 1.11)	1.02 (0.98 – 1.05)	0.97 (0.94 – 0.99)	1.00 (Ref)	1.07 (1.04 – 1.11)	1.10 (1.06 – 1.14)	1.10 (1.06 – 1.14)
**2021 - 2023**							
Tmax (ºF)	38.84	42.98	52.16	67.46	80.96	87.98	91.04
Rate Ratio (95% CI)	1.11 (1.07 – 1.15)	1.04 (1.01 – 1.07)	0.98 (0.95 – 1.00)	1.00 (Ref)	1.00 (0.98 – 1.03)	1.02 (0.99 – 1.06)	1.04 (1.00 – 1.08)

Estimated using generalized linear quasi-Poisson distributed models, with the natural log of the total number of daily scheduled visits as an offset term. Daily temperature was modeled using natural cubic splines to capture non-linear associations, adjusted for day of the week, month, year, daily precipitation, snowfall, and practice fixed effects. Data were stratified in 3-year increments to examine differences over the 15-year follow-up period.

© 2026 Mayne SL et al. *JAMA Network Open*.
