# Supplementary material for: Extreme Temperatures and Missed Pediatric Preventive Care Visits
Source: JAMA Netw Open. 2026 Apr 27;9(4):e2610114. doi: 10.1001/jamanetworkopen.2026.10114 (PMC13122397; doi:10.1001/jamanetworkopen.2026.10114)
Supplement: Supplement 2. — Data Sharing Statement [file jamanetwopen-e2610114-s002.pdf]

Data Sharing Statement

Mayne. Extreme Temperatures and Missed Pediatric Preventive Care Visits. *JAMA Netw Open*. Published April 27, 2026. doi:10.1001/jamanetworkopen.2026.10114

Data
Data available: No

Additional Information
Explanation for why data not available: The dataset includes individual-level patient health information that will not be shared in order to protect confidentiality.
